# Tracheotomy in Croup

**Published:** 1888-08

**Authors:** Nathan Jacobson

**Affiliations:** Lecturer on Laryngology and Clinical Surgery, College of Medicine, Syracuse University; 115 North Salina Street


Buffalo Medical and Surgical Journal

VOL. XXVIII. AUGUST, 1888. No. 1.

### Original Communications.

### *TRACHEOTOMY IN CROUP.**

BY NATHAN JACOBSON, M. D.,

Lecturer on Laryngology and Clinical Surgery, College of Medicine, Syracuse University.

Croup is the clinical expression of acute laryngeal stenosis. The larynx, invaded by catarrhal or diphtheritic disease, has its lumen sufficiently lessened to occasion mechanical impediment to the air-current.

As a result of temporary, local or remote excitation, we may have laryngeal spasm—laryngismus stridulus. With its subsidence, respiration at once becomes free and unobstructed. Dyspnœa from catarrhal croup, usually only a nocturnal manifestation, occasionally becomes persistent, and so severe as to excite alarm. On three occasions I have been asked by physicians to see such cases for the purpose of tracheotomizing them. In my own practice I have never had occasion to entertain the thought of tracheotomy in this class of cases.

Disregarding the identity or non-identity of membranous and diphtheritic croup in considering the advisability of tracheotomy, we are primarily concerned with the mechanical hindrance the diseased deposit or infiltration occasions, and not with its pathological character. We have to deal with the condition, and not the disease. Secondarily, however, our pathological views influence the steps of the operation as well as the after-treatment.

---

* Read at the meeting of Third District Branch of the New York State Medical Association, held at Norwich, N. Y., June 21, 1888.

Given a case of croup, in which, despite medicinal treatment, the laryngeal stenosis has progressed; hoarseness has grown to aphonia; the respiratory sounds, at first both prolonged, are now characterized by the laryngeal stridor of inspiration; in which the dyspnœa has increased, and spells of suffocation have appeared; in which the accessory muscles of respiration, called into activity by the increasing obstruction in the larynx, make the whole chest heave with their efforts to pump air into the lungs, only to have their futile efforts compensated by suprasternal, intercostal, and epigastric recessions, while the terrified child throws itself about and clutches at its throat for air—given such a case, what have we to gain by longer delaying operative interference?

Mackenzie suggests that perhaps ten per cent. of these cases recover unoperated. To me this is a more than generous statement. My experience has invariably been that, while I have seen a few cases in which the disease was arrested at the beginning of this second stage, those reaching its full development have advanced to the third, where the dyspnœa becomes intensified, the suffocation persistent instead of spasmodic, the lips and nails blue, the superficial veins, especially of the neck, greatly engorged, the lungs œdematous, the intellect dulled, until finally the comatose patient, after a frightful struggle, dies of heart failure or exhaustion.

At what period, then, shall we operate?

No one pretends that any medicinal treatment is of any avail once the third stage has been reached. Tracheotomy is now the last resort. But is it wise—is it not, indeed, cruel—to wait until the disease has all but exhausted the patient; the unærated blood has rendered him unconscious, and, with lungs engorged and heart weakened, he has but little vitality left to begin anew the struggle for existence?

More than this, the operation is much more difficult when venous congestion has buried the larynx deeply in the neck. It must be performed more hastily, is accompanied by greater hemorrhage, and the danger of fatal syncope is much increased.

Operating early, the surgeon is frequently accused, even by his considerate professional brethren, of operating unnecessarily. To wait until the patient is moribund, is certainly unjustifiable.

In attempting to establish fixed rules for the time of operation, physicians in different countries have reached varying conclusions. Renault, in his *Manuel de Tracheotmie*, published last year in Paris, tells us that in the Parisian hospitals surgeons are governed rather by the degree of permanent stridor, than the frequency or severity of the suffocative spells. If there has been progressive laryngeal stenosis for twelve to twenty-four hours, the operation is indicated. In Germany the opposite rule holds good. There, as with us, the operator is guided largely by the severity of the paroxysms and the length of the intervening periods.

In private practice the conditions differ. An intelligent attendant is not always in charge; the operator cannot be summoned at a moment's notice; the patient may be miles from his physician; night may be at hand, and every one knows how much good light simplifies the operation. Moreover, the patient may grow suddenly worse. Recently I was called to operate; the physician telephoning said that a few moments before, he had left the patient in a comfortable condition. Not a half-hour intervened, and I was at the house, only to be met at the door and told by the father that the paroxysms had become suddenly more severe, and the child had died shortly after the doctor's departure. Sanné says that one paroxysm has been severe enough to cause death; and again, it "frequently happens that the disease is unexpectedly aggravated."

If pharyngeal diphtheria has preceded the laryngeal invasion some days, and constitutional infection exists, or even if it does not, the case demands tracheotomy at a much earlier period than when the laryngeal disease has been the primary manifestation. A free supply of air counteracts the toxic influence of the disease. In this class of cases the laryngeal obstruction takes place very insidiously, and the patient is gradually accustomed to endure a lessened air supply. At the same time, the resisting power to systemic infection is correspondingly lessened.

As soon as the physician is satisfied that his efforts to control the disease are futile, it is his duty to no longer exhaust his patient with emetics, and thereby imperil the success of a future operation, but at once to relieve the asphyxia. While considering the character of the breathing, the fact cannot be too strongly emphasized that it is labored, not rapid, breathing that tracheotomy relieves. Contrary, however, to what many text-books teach, the respirations are, as a rule, increased in frequency in laryngeal obstructions (usually to about thirty per minute), for, while the respiratory act is lengthened, the intervals between the respirations are shortened, and even lost. The marked exception to this is where the laryngeal disease has slowly supervened upon the pharyngeal. Here, even, the number of respirations rarely falls below the normal. Should the respirations exceed fifty per minute, and the temperature and pulse be correspondingly high, some pulmonary complication is to be feared.

Early this month I was called out of town to tracheotomize a child afflicted with diphtheria. It had never occurred to me until then that one might mistake the snoring breathing accompanying enlarged tonsils, for that of laryngeal stenosis.

With asphyxia, requiring tracheotomy for its relief, are there any contra-indications to deter us from its performance? Extreme youth has been regarded a substantial objection by many in the profession. Dr. Winter has, however, collected ninety-three successful cases—among which is one of my own—where tracheotomy was successful in children under two years of age. While the operation is more generally successful between the fifth and eighth years, it is evidently not to be cast aside even in infants. At the very time when Trousseau was earnestly working to establish a foothold for tracheotomy, Scoutetten operated his own babe, but six weeks old, successfully. Less fortunate in adults, Sanné reports recoveries at thirty-five, forty-seven and fifty-two years of age.

Trousseau did not operate croup when secondary to measles or scarlatina, and recent writers have shared this opinion. I

have three times done the operation when measles had preexisted; in one it was successful. It must be borne in mind that laryngitis is a normal manifestation of measles during the pre-eruptive period, but that laryngeal diphtheria presents itself later, usually when the rash is disappearing. Millard had three successful cases, Sanné four, and Pilcher one. This, then, can be no contra-indication.

The difficulty of establishing the exact condition of the lungs when laryngeal stenosis exists, is generally recognized. Yet, with marked broncho-pneumonia, with œdema of the lungs from delayed operation, and indeed, with croupous pneumonia coexisting, tracheotomy—though the prospect is not hopeful—has resulted happily many times.

If I were to establish any contra-indication, it would be the simultaneous existence of malignant pharyngeal diphtheria. And yet even here, though death probably will result, the breathing is rendered so free that we feel the force of Dr. West's statement: " Many of the deaths after tracheotomy are, strictly speaking, far from being instances of failure."

Poor hygienic surroundings, the lack of proper assistants, no prospect of good nursing, may cause us to hesitate, but even under such circumstances I have seen the operation succeed. Nothing ever impressed me more forcibly than when, as a student of the late Dr. R. W. Pease, of Syracuse, I was permitted to be with him as he reached a house where the little child, the father said, had but that moment died of croup. Without an instant's delay the doctor cut down upon the insensible neck, reached the trachea, placed his tube, and resorted to artificial respiration. Patience and perseverance soon resulted in independent breathing. The child lived three days after this, a lasting proof of the possibilities of this operation.

It is, indeed, an excellent indication of the popular appreciation of the benefits of tracheotomy, when a parent solicits the operation at the hands of his physician. It is the triumph of medical progress when a parent, refused by the physician in attendance—who has no faith in tracheotomy—runs from one physician to another, meeting excuses here and refusal there, persistent in his demands, until he brings to the bedside a surgeon who will operate. Such was a recent experience in our city. Unfortunately, we are called to these cases to find the patient moribund, no time for preparation, no assistants. But we operate.

Where you can, select a narrow table, cover it with a quilt and sheet; place it where the light will fall upon the child's neck. Station one assistant at the head of the table to administer the anæsthetic, another at the left to sponge and hand you the instruments, and yourself at the right. I have invariably used chloroform; it is to be administered slowly. The sudden exposure of the child to it may excite increased asphyxia; under its influence, respiration becomes easier. Sanné thinks it may usually be dispensed with after section of the skin, and Gill, who has translated his work, argues that he has seen deaths attributable to its use. Once I saw it act unfavorably. The dyspnœa became intensified; I discontinued it, and completed the operation without an anæsthetic. The tube was placed, unobstructed breathing had been established, when suddenly the child died. I have always attributed the death to cardiac paralysis, rather than to the chloroform. I have never carried anæsthesia to the point of rendering the trachea insensible to the presence of blood or mucus.

The patient anæsthetized, a rolling-pin, covered with a towel or a small cushion, is placed under the shoulders, and the head thrown back. In securing this position—known as Rose's—care should be exercised not to approximate the anterior and the posterior walls of the trachea too closely by using too large a cushion. The neck is now to be thoroughly cleansed, for usually the solicitous friends have employed various external applications. The line of incision depends upon the operation selected. The thyroid isthmus in the adult covers the second and third tracheal rings, and forms the boundary line between what English surgeons recognize as the high and low operation. The limited space between the cricoid cartilage and the thyroid isthmus, the danger of incising the latter and provoking a perfect wave of blood, the fact that the isthmus, especially in children, frequently lies directly over the cricoid, accounts for the unpopularity in this country of an incision into the trachea at this point. Abroad, the superficial location of the laryngo-tracheal region has induced most recent authorities to favor high tracheotomy, and even crico-tracheotomy.

I have always proceeded as follows: With the index finger of the left hand, I locate the central prominent tuberosity of the cricoid. Beginning at this point, an incision not less than an inch in length is carried downwards in the median line. It is needless to indicate its course with a pencil or ink, as has been suggested. I believe it unwise to adopt the advice of Renault of fixing the larynx between thumb and finger, or of using for the same purpose a tenaculum, as Chassaignac and Langenbeck suggest, as any effort to restrict the free movement of the trachea favors the increase of asphyxia. After the incision has been carried through the skin, if a scalpel has been used, a bistoury is to be substituted. For it has occurred that the prominent belly of the knife, especially in young children, has depressed the trachea and failed to penetrate it. All further cutting is to be done with the point of the knife. The operation is to proceed carefully but not too slowly. The incision is not to be funnel-shaped, but in its depth nearly as long as at the surface. Rapid, sweeping incisions are demanded only in extreme cases, but timid, uncertain ones create little pockets in which infectious matter may become lodged, and, later, excite cellulitis. A fatal case of this kind is credited as my own, although I merely completed the operation after it had advanced to this stage in the hands of another.

Retractors, tenacula, forceps to separate the edges of the incision, are too apt to lead the operator from his proper course, even to the cervical vertebræ. The extremity of the index finger is the most reliable guide. With it the trachea can be constantly felt, its depth from the surface ascertained; and what is more, the operation proceeds without regard to the amount of bleeding, and should any abnormal distribution of the innominate artery or any other large vascular branch exist, its detection is most certain.

When the tracheal rings can be felt, provided there is no arterial hemorrhage, we are ready to perforate. Venous bleeding will not cease until the asphyxia causing it is relieved. With the edge of the knife directed upwards, incise the trachea. It is the most trying moment of the operation. Not infrequently the patient ceases to breathe. There is apparent death. No moment is to be lost. The trachea is not to be nicked here and there; but guided by the finger, a central incision is to be made, and as soon as the air is heard to hiss through the blood in the wound, a blunt bistoury is to be substituted and the incision carried up through two or three rings. The three-bladed dilator is now at once introduced. In a moment the child inspires deeply, coughs out the blood which has been sucked into the trachea, the venous hemorrhage is checked and the danger is passed.

I know that these steps do not accord with those recommended by the German and French authorities. The latter tell us that with the larynx fixed between the thumb and middle finger of the left hand, the index finger remains in the wound. The sharp bistoury having penetrated the trachea is at once carried upwards, and the incision is completed without the aid of the blunt bistoury. Now, unaided by a dilator, the index finger still guarding the tracheal orifice, the canula we are told can easily be inserted. Dilators take up too much room; the change of bistoury too much time. As not a few times the sharp-pointed bistoury has cut its way into the œsophagus, the change to the innocent blunt one is unquestionably wise.

Are we ready at once for the canula? Most certainly not! The trachea is occupied by mucus, septic matter, and perhaps, like the larynx, also with false membrane, which, as we know, presents varying anatomical characteristics as it fades from a membranous deposit into a layer of pus. These foreign substances must be removed; as a rule they are coughed out. Should they not be we are to effect their removal, not by suction with the mouth, nor with the aspirator of Parker, which has a mouthpiece, but by means of a catheter carried into the trachea and attached to a bulb syringe. If the substances are not easily dislodged a spray can be used. I prefer a solution containing one drop of liq. potassæ to an ounce of lime water ; it possesses remarkable solvent properties. It matters not, however, what is used, provided the object is attained.

The importance of this step I saw recently illustrated in a case of secondary diphtheria following upon capillary bronchitis, in a child who had just had measles. After the trachea was opened the asphyxia remained unrelieved. Had the canula been immediately introduced nothing would have been gained. With catheter and syringe we literally pumped out the little girl. In this case the diphtheritic pus accumulated so rapidly that it was found necessary to introduce a large canula, through which a Jacque's catheter was carried every three or four hours into her large bronchial tubes, and the matter withdrawn after the manner indicated.

Moreover, diphtheritic accumulations are carried from the upper air passages into the lungs, and obstructing the bronchioles, excite broncho-pneumonia. I have no reason to doubt Sanné's statement that, in Paris, broncho-pneumonia is the scourge of the tracheotomized, when we know that they overlook the existence of this foreign substance, capable of infecting the lungs. Having cleared the channel we are ready for the introduction of the canula. Any one who has attempted this unassisted by a dilator, knows how difficult is the task. The three-bladed dilator is the most useful instrument at our command. The two-bladed instrument creates only a transverse slit; the third blade converts this into an opening. It is true that in small larynges considerable space is occupied by the dilator. For this reason, the blades of this instrument should be extremely thin.

The complicated guides, such as Lisfranc and others have invented, have occasioned more harm than good. Should too small an opening exist it is to be enlarged. If a non-median incision has been made it is to be disregarded, and a new one substituted. The utmost gentleness must attend this part of the operation. Any force is apt to produce a false route—the starting point of an abscess—leading even to the mediastinum.

In this country we use the quarter circle tubes. Robert W. Parker in his able work on "Tracheotomy and Laryngeal Diphtheria," argues well for the angular canula. The instrument, preferably metallic, is, of course, to be double. The inner tube should project just a little beyond the outer. As the calibre of a tube is equal only to its smallest diameter, tapering tubes are a disadvantage. For obvious reasons the largest size the trachea will endure should be selected. The plate should allow sufficient play for the tube to meet the movements of the neck; its ends should be perforated. The tapes should not be indirectly attached to it through wire eyelets, as the free movement thus permitted may excite gangrene of the wound, and even erythema of the chest. While the dilator is being removed, the canula should be steadied in its place until the tapes are fastened, that it may not be coughed out. The tapes can be tied moderately tight, as the circumference of the neck is speedily reduced. Care is to be exercised that the tube has not been simply forced between membrane and tracheal wall. Should the wound be too long, a suture is to be taken below. A piece of oiled silk is carried under the plate and around the tube to protect the wound from infectious discharges. The tube opening is now to be covered with tulle and the child placed in a croup bed. The bed is readily extemporized. To the four corners of the bed upright strips can be fastened, and these mounted with transverse ones to hold them together. If made to project a little in front of the bed, room is given for a steam-producing appliance. Sheets are now thrown over the frame-work and draped back in front. An atmosphere of steam is created and the temperature maintained at seventy-five to eighty degrees. In this bed the little patient is sufficiently protected, so that the room in which it is placed can be properly ventilated.

Constant, unremitting, watchful care is necessary; an attendant must be ever at hand to meet any emergency that may arise.

The operation has established a channel for respiration; this must be kept pervious. I believe in the faithful use of a spray directed to the opening of the canula, using the alkaline solution already mentioned, a five per cent. solution of papoid, or some other good solvent. Additionally, I employ a disinfectant spray of one-half to five per cent. of carbolic acid, as Jacobi recommends, one of borax and salicylic acid or, preferably, a solution of potass. permanganate, two grains to the ounce.

It must not be lost sight of that tracheotomy is not a remedial measure. It does not influence the disease further than to supply air to the lungs. The disease is ever present and requires as much, if not more, attention. The pharynx is not to be neglected; the infectious sites must be sprayed at least hourly. It is not the material used, but the method of its employment, which is essential—faithful, persistent, local disinfection. Not less necessary is it to destroy all infected clothing, and to disinfect every vessel used.

The general condition is to be improved with tonics, the generous supply of stimulants, concentrated nourishing foods, milk, egg-nog, beef juice, and the like. Mental quiet, constant rest in bed, lest some excitement may awaken fatal syncope, is to be enjoined. The inner canula is to be removed when necessary. It is also to be cleansed in an alkaline, and purified in a disinfectant solution. A fluttering against the lower extremity of the canula indicates the presence of membrane or viscid mucus. If, with the solvent spray, these are not removed the attendant should be able to remove the canula, and with forceps or otherwise, remove the obstructing masses.

Our French *confrères* believe in removing the double canula daily, and, after allowing the patient to do without it for an hour or two if possible, to replace the tube or substitute another. I can see no reason for this step earlier than the third day unless specially indicated. Marked disturbance of the wound soon manifests itself, and with cleanliness and disinfectants I have rarely seen this occur. Moreover, as Bartels shows, the frequent changing of the tube irritates the wound and the trachea.

When, with the tube out and the wound closed, the child is able to blow out a lighted candle, the time for the final removal of the canula has arrived. In the majority of cases this can be done about the eighth day. In my earlier cases, perhaps because of my timidity, I allowed it to remain much longer. The urine is to be closely watched, the temperature taken, and the expectoration observed. Unless some complication arises the latter loses its bloody character after twenty-four hours. When there is no discharge the prognosis is unfavorable. The same precautions are to be taken, whether or not pharyngeal diphtheria existed or co-exists. The patient is always to be isolated from the other children in the family, if possible. The absence of pharyngeal disease does not guarantee immunity from constitutional infection. In one case seen in the practice of another physician—who is a firm believer in the non-identity of membranous and diphtheritic croup, without any pharyngeal manifestations, any co-existing cases of diphtheria—his patient died of uremic convulsions six days after tracheotomy. In another a medical student toyed too long with the membranous discharges, and the absence of pharyngeal disease did not prevent his infection. Nor is it certain that all children exposed to diphtheria will become infected. In the last case, to which a *confrère* called me to operate, a girl five years old, sick for ten days with severe pharyngeal diphtheria, gradually extending into the larynx, the urine so rich in albumen at the time of operation that it entirely coagulated under heat, and who died, four and a-half days after operation, of paralysis of the pneumogastric; a younger brother and sister hovered about her constantly, yet neither acquired the disease. This last instance, where the family were compelled to keep the children at home and where it was impossible to separate them from the invalid, is not introduced to justify the measure. But these cases emphasize the fact that upon the presence or absence of pharyngeal manifestations, we should not base our opinion of the infecting or non-infecting character of primary laryngeal disease.

Should complications arise, they should be closely watched and treated. When the tube has been removed a dry dressing of iodoform or sublimated gauze, with or without strapping, is to be applied. Several weeks should elapse before the child is allowed to mingle with its playmates.

My own experience covers twenty-two tracheotomies; seven_teen of these were done for acute laryngeal disease. Of the remaining five, two were performed for laryngeal syphilis, one presumably for the removal of a foreign body from the larynx, but, as it developed, for peri-chondritis of the tracheal cartilages; one preceding resection of the superior maxilla for the removal of a naso-pharyngeal tumor, and, finally, one on a child but seventeen months old, in whose right bronchus a bean had become impacted, the details of which can be found in the *Medical Record*, December 30, 1882. Each of these five patients recovered. It is evident that I have no reason to fear tracheotomy in itself. I believe it to be an operation whose dangers are over-estimated. Of the remaining seventeen, and, as stated, the list includes one fatal case begun by another surgeon and completed by myself, five recovered; nine had pharyngeal diphtheria; in three, the laryngeal disease was secondary to measles, and in only five was there uncomplicated primary laryngeal diphtheria. The youngest operated were fourteen, seventeen, twenty-three, and two twenty-four months old. Of this number, the one aged seventeen months recovered. No case could have been surrounded with more trying circumstances: the mother dying of consumption a few days after operation; the father continually under the influence of liquor; extreme poverty; a wretched tenement-house; only our kindly-disposed medical students to give her any care, and, finally, the removal of the child, during the bleak days of March, to St. Joseph's Hospital, Syracuse. Of the next five, one was three, and four were five years old; of these, one of the last recovered. The rest were respectively, one, six; three, seven, and one eight, nine and ten years of age; of these, three recovered.

As to the causes of death, one died immediately after the operation of cardiac paralysis; three of pulmonary œdema, due to the delay of operation, twenty-two to forty hours after its performance; one babe of cholera infantum, twenty-eight hours, another probably of meningitis, thirty-six hours; and a third of high temperature, forty-eight hours after operation. Of the remainder, one died of cellulitis two and a-half days; one of exhaustion four days; one of diphtheritic paralysis four and a-half days; and one of uremic poisoning six days after operation. In the successful cases the tube was removed twice on the eighth, twice on the seventeenth day, and once its removal was delayed to three months. With a single exception none of these seventeen cases occurred in my own practice; in each case other physicians had exhausted all means of securing relief, and tracheotomy was performed as the *dernier ressort.*

I prefer to state that in all of these cases, done to overcome mechanical hindrance, which threatened death by suffocation, tracheotomy was a success, and that not merely thirty per cent. of my cases had a favorable termination. The statistics found in the text-books do not state the matter forcibly enough. As the operation is done when, without it, the issue must be fatal, a single successful case justifies it, and a thousand deaths after its performance do not invalidate this claim. There remains but one other question: Have we any substitute for tracheotomy in croup?

From the time of Hippocrates, until Asclepiades suggested the operation of tracheotomy, physicians had attempted the introduction of an elastic tube into the larynx through the nose or mouth. The cutting operation, as we can readily understand, entirely superseded this procedure, until early in the present century, Desault, as Ryland wrote in the Jacksonian prize essay in 1835, in consequence of the unfortunate issue of two or three unsuccessful tracheotomies, "strongly urged the introduction into the trachea of an elastic tube by way of the nostrils." Again it failed to impress the profession.

After Trosseau had firmly established tracheotomy in France, Bouchut urged intubation of the larynx, suggesting the use of a metallic tube. But, as Jacobi writes, " His enthusiastic praises of that method have contributed more than its deficient success to its speedy downfall. For not only did he claim complete comfort and relief from dyspnœa for the procedure, but instant restoration of voice."

During the past three years, through the earnest efforts of O'Dwyer, laryngeal intubation is again before us as a substitute for tracheotomy. I have attempted the operation twice, and in each ease the distress was aggravated, for the tube became quickly occluded with viscid muco-pus, and after several unsatisfactory attempts with the O'Dwyer tube, tracheotomy had to be performed.

The latest statistics at my disposal (*Sprengel Centralblatt der Chirurgie*, June 2, 1888,) covers a record of 806 intubations in the hands of sixty-five different physicians, of which 221, or 27.4 per cent., recovered. In the successful cases the average length of time in which the tube was worn was five days and three and one-half hours. Of the 585 fatal cases the cause of death was indicated in 339. Of these, 139 died of diphtheritic bronchitis, fifty-five of pneumonia, thirty-seven of sepsis, thirty-nine of exhaustion, twenty-five of nephritis, twenty of heart failure, nine of œdema of the lungs, seven of bronchitis, two of asphyxia from plugging of the canula, two of asphyxia from membrane carried into the larynx from above; one of asphyxia from swelling about the tube, and four from coughing out the tube.

It appears from these figures that intubation, after the O'Dwyer method, has not been blessed with a larger percentage of successes than tracheotomy. The comparison of the statistics of the two methods is obviously unprofitable, for the general conditions co-existing with those demanding operation, the surroundings of the patient, his age, the existence of tracheal disease, and the many other conditions which must be weighed, are obviously of too much importance to permit the determination of the value of either procedure by mere mathematical calculation.

However, intubation in its improved form has been long enough before the profession to permit an estimate of its real value and an appreciation of its dangers. While it is not accompanied by the same degree of shock as tracheotomy, there attends intubation, performed as it is, while the child is struggling in the nurse's arms, a certain element of danger from fatal syncope. If tracheotomy requires a skillful operator and able, available attendants after operation, the statement applies with even more force to intubation, where expertness of the physician is in greater demand in moments of danger after the tube has been placed—for its removal is much more difficult than its introduction. The one patient can safely be entrusted to a good nurse; the other demands the close attendance of a surgeon, for the tube may be suddenly occluded or coughed up. Tracheotomy permits the removal of infectious matter; intubation not only does not, but in some cases pharyngeal deposits have been carried with the tube into the larynx, an accident which has, at times, demanded immediate tracheotomy. All this accounts for the much greater number of deaths from pulmonary infection after intubation.

The criticism of Sprengel, above referred to, includes the statement of fifteen objections to intubation, frankly stated by Dillon Brown, one of its most earnest advocates.

It is unnecessary, in this paper, which has already greatly exceeded the length I intended it should, to consider these. I simply quote the critic: "According to the foregoing statistics, which are decidedly more unfavorable than those obtained in more recent times by tracheotomy, and also because of the many dangers which attend intubation, the discoverer of this method need not be astonished if the procedure has not encountered the same enthusiasm everywhere which it has awakened in America."

And yet there is no doubt that, in a limited number of cases, intubation will be found serviceable, especially in very young children, those under the age of three years, in the cases where the disease is well up in the larynx, where none of the pultaceous deposit exists found in the lower respiratory track, and in a large number of cases where parents will not yield to a cutting operation.

I cannot believe that these two operations are to be regarded as rivals, applicable to the same classes of cases. I prefer to consider them help-mates, each meeting the individual cases for which it is best suited.

ADDENDA.

Since writing the above, the proceedings of the last Congress of German Surgeons have come to hand. At this meeting, Thiersch, of Leipsic, presented the subject of intubation. He exhibited the table of Dillon Brown containing the 806 cases, upon which Sprengel's criticism is based.

During the past ten years 697 tracheotomies have been performed at the Leipsic clinic, of which 24.5 per cent. recovered. During the first three months of this year intubation was given a trial. Thirty-one children were intubated, of which seventeen subsequently required tracheotomy. The cutting operation was performed in eleven for respiratory difficulties; of these, seven had slowly-increasing dyspnœa, and four were threatened with suffocation from sudden plugging of the tube with membrane. In the remaining six the trouble was with deglutition. None of the seventeen recovered; nine died of pneumonia, one each of septic diphtheria, nephritis and pulmonary gangrene, and five of several of these conditions combined.

Of the remaining fourteen, while the dyspnœa was relieved in each case, eleven died; three of pneumonia, three of septic diphtheria, two of nephritis, and three of a combination of these causes. Three recovered; one after the canula, filled with tube casts, had twice been coughed out, and two who had repeatedly cast off thin shreds of membrane. Thiersch refers to the absence of irritation in many cases where, without difficulty, the tubes were worn for six days and longer.

As in Dillon Brown's table no mention is made of slowly-increasing dyspnœa after intubation, and of only five instances of asphyxia from membranous collections, Thiersch argues that we must have a milder variety of diphtheria in America than they encounter in Leipsic.

In his summary the writer says that in Leipsic the most brilliant success of intubation was in its primary results. He considers it a substitute for tracheotomy when the membranous formations and swelling are moderate. It is applicable without professional assistance; the consent of parents is more easily gained; there is no hemorrhage, wound infection, tracheal granulations and stenosis; and no marked injury from the canula was observed. Yet it is possible to bruise the mucus membrane and excite infection when the introduction or removal of the tube is difficult. The great danger of sudden suffocation indicates the need of constant medical attendance. When the epiglottis or tracheal mucus membrane is swollen, intubation is useless. Broncho-pneumonia, and even inanition, are frequent results of the difficulty of swallowing. The future of intubation in laryngeal diphtheria in Germany will depend upon increasing the lumen of the tube, the facilities offered for its immediate removal when suffocation threatens, and overcoming the impediment to deglutition.

Rehn, of Frankfort, also contributed an article upon intubation. While, he says, O'Dwyer has presented a much-improved method, intubation cannot displace tracheotomy. After considering the details of its application he argues that, because of their need of skillful after-treatment, intubated patients can be cared for properly only at a hospital. Of his fourteen cases only one was able to swallow soft foods. The others strangled so that he was obliged to resort to nutrient enemata, the œsophageal tube, and even to remove the intubation tube in some cases whenever food was given.

Both Thiersch and Rehn appreciate the conscientious work O'Dwyer has performed, the desirability of a bloodless substitute for tracheotomy, and trust that, as the profession acquaints itself with the subject of intubation, its present objectionable features and difficulties may be surmounted.

115 North Salina Street.

